# New evidence of arsenic translocation and accumulation in *Pteris vittata* from real-time imaging using positron-emitting ^74^As tracer

**DOI:** 10.1038/s41598-021-91374-1

**Published:** 2021-07-08

**Authors:** Yi Huang-Takeshi Kohda, Zhaojie Qian, Mei-Fang Chien, Keisuke Miyauchi, Ginro Endo, Nobuo Suzui, Yong-Gen Yin, Naoki Kawachi, Hayato Ikeda, Hiroshi Watabe, Hidetoshi Kikunaga, Nobuyuki Kitajima, Chihiro Inoue

**Affiliations:** 1 Graduate School of Environmental Studies, Tohoku University, Miyagi, 980-8579 Japan; 2 Faculty of Engineering, Department of Civil and Environmental Engineering, Tohoku Gakuin University, Miyagi, 980-8537 Japan; 3 Research Institute for Engineering and Technology, Tohoku Gakuin University, Miyagi, 980-8537 Japan; 4 Takasaki Advanced Radiation Research Institute, National Institutes for Quantum and Radiological Science and Technology (QST), Gunma, 370-1292 Japan; 5 Cyclotron and Radioisotope Center (CYRIC), Tohoku University, Miyagi, 980-8578 Japan; 6 Research Center for Electron Photon Science (ELPH), Tohoku University, Miyagi, 982-0826 Japan; 7 Technology Development Division, Fujita Corporation, Kanagawa, 243-0125 Japan

**Keywords:** Plant sciences, Environmental sciences

## Abstract

*Pteris vittata* is an arsenic (As) hyperaccumulator plant that accumulates a large amount of As into fronds and rhizomes (around 16,000 mg/kg in both after 16 weeks hydroponic cultivation with 30 mg/L arsenate). However, the sequence of long-distance transport of As in this hyperaccumulator plant is unclear. In this study, we used a positron-emitting tracer imaging system (PETIS) for the first time to obtain noninvasive serial images of As behavior in living plants with positron-emitting ^74^As-labeled tracer. We found that As kept accumulating in rhizomes as in fronds of *P. vittata*, whereas As was retained in roots of a non-accumulator plant *Arabidopsis thaliana*. Autoradiograph results of As distribution in *P*. *vittata* showed that with low As exposure, As was predominantly accumulated in young fronds and the midrib and rachis of mature fronds. Under high As exposure, As accumulation shifted from young fronds to mature fronds, especially in the margin of pinna, which resulted in necrotic symptoms, turning the marginal color to gray and then brown. Our results indicated that the function of rhizomes in *P. vittata* was As accumulation and the regulation of As translocation to the mature fronds to protect the young fronds under high As exposure.

## Introduction

Arsenic (As) is a toxic element that is usually found in soils and groundwater as a result of both natural and anthropogenic processes. Chronic exposure to inorganic As may lead to cancers of prostate, bladder and skin^1^. *Pteris vittata* is the first fern to be identified as an As hyperaccumulator that can accumulate As to > 22,000 mg/kg dry weight in fronds when grown in soil spiked with As at 1500 mg/kg^2^. As concentration in contaminated water can also be reduced by *P*. *vittata* to a level that is within the World Health Organization (WHO) guidelines^3^. Different from other remediation methods, phytoremediation of As-contaminated soils and water by *P*. *vittata* is more environmentally friendly. *P*. *vittata* is a unique model to study the interactions between plant and As.

Arsenate (AsV) and arsenite (AsIII) are two major inorganic As species in water, with AsV the more predominant form in aerobic water. In *P*. *vittata*, AsV is taken up by roots via phosphate transporters^4,5^, and then AsV is translocated from roots to rhizomes and reduced to AsIII^6,7^. Different from other plants, rhizomes are the unique underground stems that connect the roots and fronds in fern plants like *P*. *vittata*^8,9^. In many studies rhizomes and roots in *P*. *vittata* are not separated and are both recognized as roots^10,11^. Thus, there is little available information on As accumulation in rhizomes of *P*. *vittata*.

As (AsV and AsIII) in rhizomes is translocated to the fronds through the xylem in *P*. *vittata*^12^. In fronds, As accumulation and distribution have been well studied by several methods, including scanning electron microscope with energy dispersive X-ray spectrometer (SEM–EDX) and synchrotron X-ray microprobe analysis. These studies found that As was concentrated at the tip of the apical pinna and decreased toward the basal pinna of fronds^13^. Inside the pinna, As was found in the proximity of veins in the pinna surface, and most of the As was localized near the midrib, which indicated that As in the pinnae is contained in the apoplast rather than vacuoles^14^. Necrotic symptoms in the pinna margin were induced by high As exposure, and the As concentration in the pinna margin was 2.3 times higher than that in the midribs^15^. These techniques only provide static data and because of the detection limit, only high As exposure experiments were conducted. Therefore, As accumulation and distribution in fronds of *P*. *vittata* under various As conditions are still unclear.

The positron-emitting tracer imaging system (PETIS) is one of the most advanced radiotracer-based imaging methods available today; its principle is the same as that of positron emission tomography, which has been widely used for medical diagnosis^16^. PETIS is specially designed for studying plants and provides serial time-course images (i.e., animation) of the two-dimensional distribution of a radiotracer within a living plant without contact; it has been applied to many studies on plant nutrition over the last decade^17^.The transport of metals in plants, including Fe^18^, Mn^19^, Zn^20,21^ and Cd^22,23^ have been visualized using PETIS. As transport has not yet been visualized in the plant by PETIS.

In this study, we investigated As accumulation in rhizomes, and As accumulation in different stages of fronds in hydroponically grown *P*. *vittata* with high As exposure (30 mg/L) in a long term phytofiltration experiment. We successfully visualized As translocation and accumulation in living *P. vittata* from low to high As exposure by using PETIS and autoradiography with positron-emitting ^74^As labeled tracer and compared it with that in the non-accumulator model plant *Arabidopsis thaliana*. This study provides important insights into the behavior of As in plants as well as providing significant information to advance our understanding of As hyperaccumulation.

## Results

### Long term As phytofiltration with *P*.* vittata*

**Figure 1:**
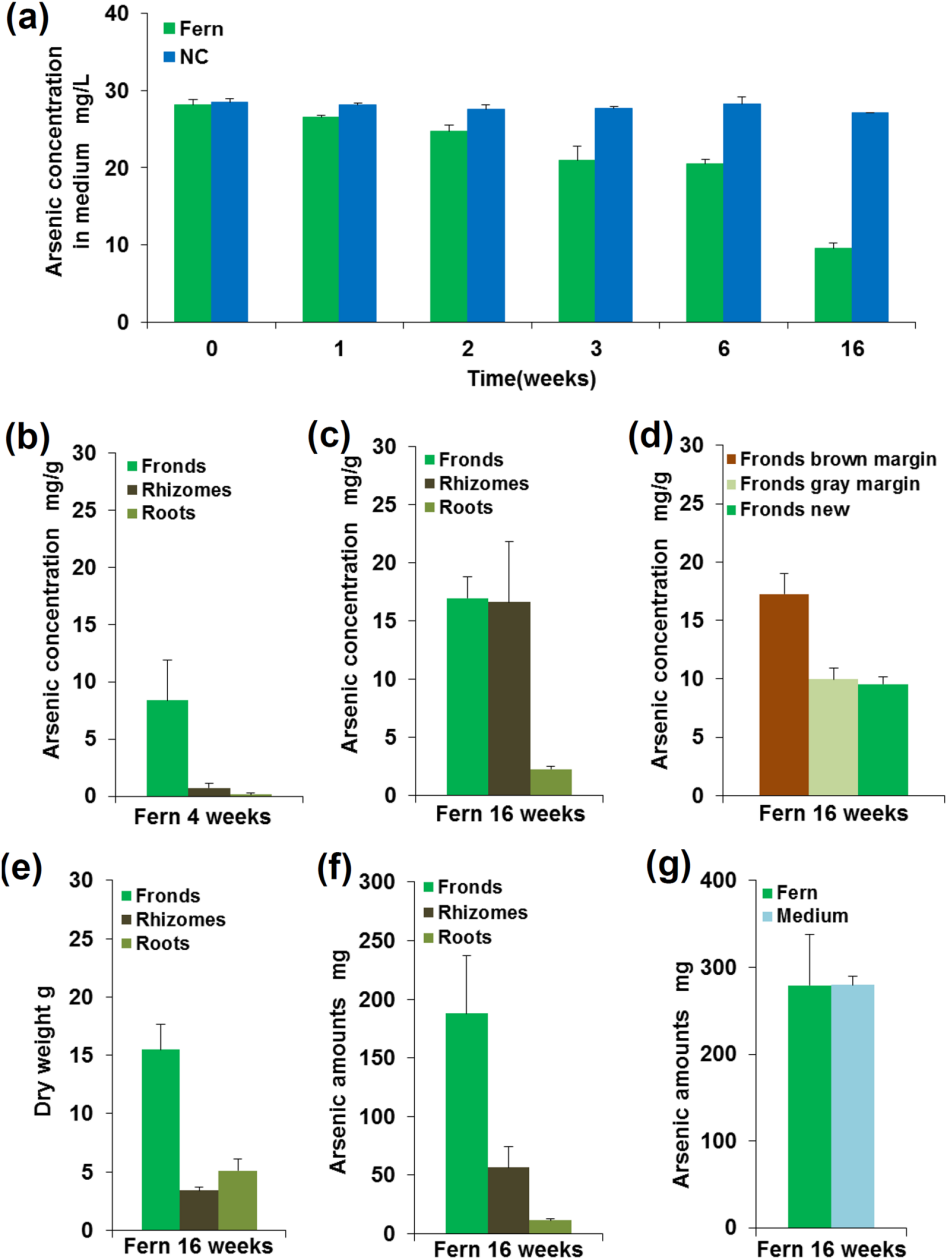
As phytofiltration experiment with *P*. *vittata* over 16 weeks. (**a**) Time course of As removal in medium with *P*. *vittata* (Fern) and without *P*. *vittata* (NC) over 16 weeks. After 4 weeks As phytofiltration experiment, (**b**) As concentration in fronds, rhizomes and roots of *P*. *vittata*. After 16 weeks As phytofiltration experiment, (**c**) As concentration in fronds, rhizomes and roots of *P*. *vittata*. (**d**) As concentration in different stages of fronds (fronds brown margin, fronds gray margin, fronds new) of *P*. *vittata*. (**e**) The dry weight of fronds, rhizomes and roots of *P*. *vittata*. (**f**) As amounts in fronds, rhizomes and roots of *P*. *vittata*. (**g**) As mass balance between As uptake by *P*. *vittata* and As decline from the medium. Data are expressed as mean + standard deviation (n = 3).

To understand the As accumulation in rhizomes and fronds of *P. vittata* with high As exposure, *P. vittata* was grown hydroponically for 16 weeks in phosphate (Pi) free hydroponic medium with 30 mg/L AsV. Within 16 weeks more than 60% of the AsV was taken up by *P*. *vittata* (Fern) when compared with the negative control (NC) (Fig. 1a). The image of the *P*. *vittata* shows that after 1 week the margin of pinnae became gray, and after 4 weeks the gray margin turned to brown and continued browning through to 16 weeks (Supplementary Fig. S1a). This finding was similar to those of several studies, but they only recorded the tissue necrosis of the brown margin pinna and ignored the gray margin pinna^15,24^. After 4 weeks, three plants were sampled and separated into fronds, rhizomes and roots; the As concentration in fronds was much higher than those in the rhizomes and roots (Fig. 1b) (*p* < 0.05). After 16 weeks the rest of the plants were sampled and separated into fronds, rhizomes and roots. When compared with plants at 4 weeks, the As concentrations in the 16-week plants were comparable between fronds and rhizomes (around 16,000 mg/kg) and much higher than that in the roots (Fig. 1c) (*p* < 0.05). This result showed that rhizomes and fronds accumulated the high levels of As, which differed from the results of a previous study that showed that the only function of rhizomes was AsV reduction and AsIII translocation to fronds^8^. The fronds were then separated into three different types: fronds brown margin (tissue necrosis), fronds gray margin (tissue necrosis) and fronds new (Supplementary Fig. S1b, c). The As concentration in fronds brown margin was higher than those in fronds gray margin and fronds new (Fig. 1d) (*p* < 0.05). Our results were supported by a previous study of high As concentration soil cultivation with *P*. *vittata*^15^, but our study showed a significant difference between fronds brown margin with fronds gray margin and fronds new, and much higher As concentrations in the fronds because the availability of As in hydroponic cultivation was higher than that in soil cultivation. On a dry weight basis, more than 95% of As was accumulated in fronds and rhizomes (Fig. 1e,f). The mass balance of As in plants and the medium was calculated; the As accumulated in the plant was the same as the declined in As observed in the medium (Fig. 1g). Our data indicated that the function of rhizomes in *P*. *vittata* was not only AsV reduction and AsIII translocation but also As accumulation, and the necrotic symptoms in the margin of pinna might be the key to high As accumulation in fronds of *P*. *vittata*.

### Seven days of PETIS imaging for As translocation and accumulation in *P*.* vittata* and *A*.* thaliana*

As translocation and accumulation in *P*. *vittata* fed with modified Pi free 1/5 Hoagland solution was real-time imaged using PETIS. Unlike the long-term hydroponic experiments in Fig. 1, PETIS imaging was conducted over 7 days. Thus, to reproduce the phenomenon of the necrotic symptom margin of pinna in fronds of *P. vittata* within 7 days, *P. vittata* were exposed to a high As concentration of 2000 μM was applied for to conduct PETIS imaging. Lower As conditions (0.2, 20 and 200 μM AsV) were also tested.

**Figure 2:**
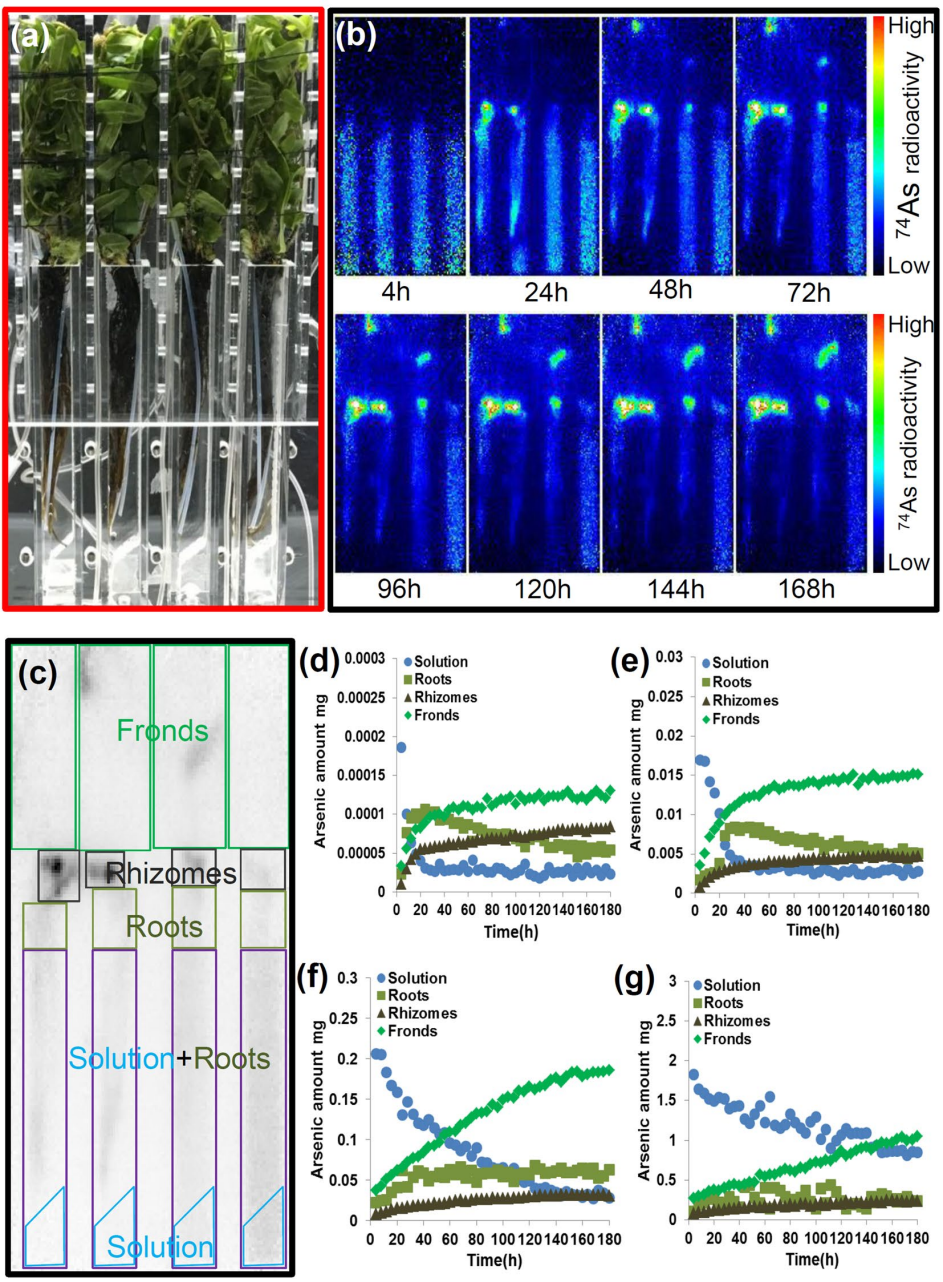
Serial images of ^74^As behavior and time course of the amount of arsenic in different regions of *P*. *vittata*. (**a**) Photograph of test plants in the experimental apparatus. The solid red rectangles indicate the field of the PETIS imaging. (**b**) Serial images of *P*. *vittata* (0–168 h). Each frame was created from the integration of original images collected every 5 min. (**c**) Examined regions in the plants. The blue trapezoid indicates the region of the solution, the purple rectangle indicates the solution with the roots inside, the yellow-green rectangle indicates the roots above the solution, the black rectangle indicates the rhizomes and the green rectangle indicates the fronds. Time course of the amount of As in the regions shown in (**c**) fed with 0.2 μM AsV (**d**), fed with 20 μM AsV (**e**), fed with 200 μM AsV (**f**) and fed with 2000 μM AsV (**g**), all experiments included 2 kBq of ^74^As-labeled AsV.

Figure 2a shows four plants supplied with 0.2, 20, 200 and 2000 μM non-radioactive AsV (total As of 0.00019, 0.017, 0.21, 1.83 mg, respectively) including 2 kBq ^74^As-labeled AsV (0.0074 pmol) and subjected to PETIS for 7 days (168 h). Figure 2b and Supplementary video S1 show representative results of PETIS imaging of As translocation and accumulation in *P*. *vittata*. Time courses of the radioactivity of ^74^As over time within each region of interest (ROI) were generated through manual selection of ROI on the image data (Fig. 2c). The ROI of rhizomes and fronds were observed as the bright spot in the PETIS imaging after 168 h, which showed that As accumulation in *P*. *vittata* occurred in both fronds and rhizomes (Fig. 2b,c). Representative curves of AsV dynamics in the solution and As uptake into the roots and translocation to the rhizomes and fronds were shown in Fig. 2d–g. Over the 168 h of experiment, the volume of solution was maintained automatically by supplying the solution without AsV, therefore AsV absorption by roots would have decreased the amount of AsV in the solution. Therefore, the curve of AsV depletion in the solution directly reflected root absorption. It was clearly showed that in the 0.2 μM As treatment, all the supplemented As in the hydroponic solution was taken up by the roots and translocated to rhizomes and fronds within 24 h after As exposure and the As concentration in the solution decreased simultaneously. It took 48 h and 144 h to observe similar depletion of the As concentration in the solution in 20 μM and 200 μM As treatments, respectively. After 168 h of the exposure to 2000 μM As, some As remained in the solution. In the 0.2 and 20 µM treatments, As in roots was gradually decreased, corresponding to As translocation to rhizomes and subsequent translocation to fronds. However, As accumulation was not decreased in rhizomes like roots and reached a plateau after 24 h, with little increase after this time (Fig. 2d–g). This result is consistent with that of the phytofiltration experiment (Fig. 1c) which showed As accumulation in rhizomes.

**Figure 3:**
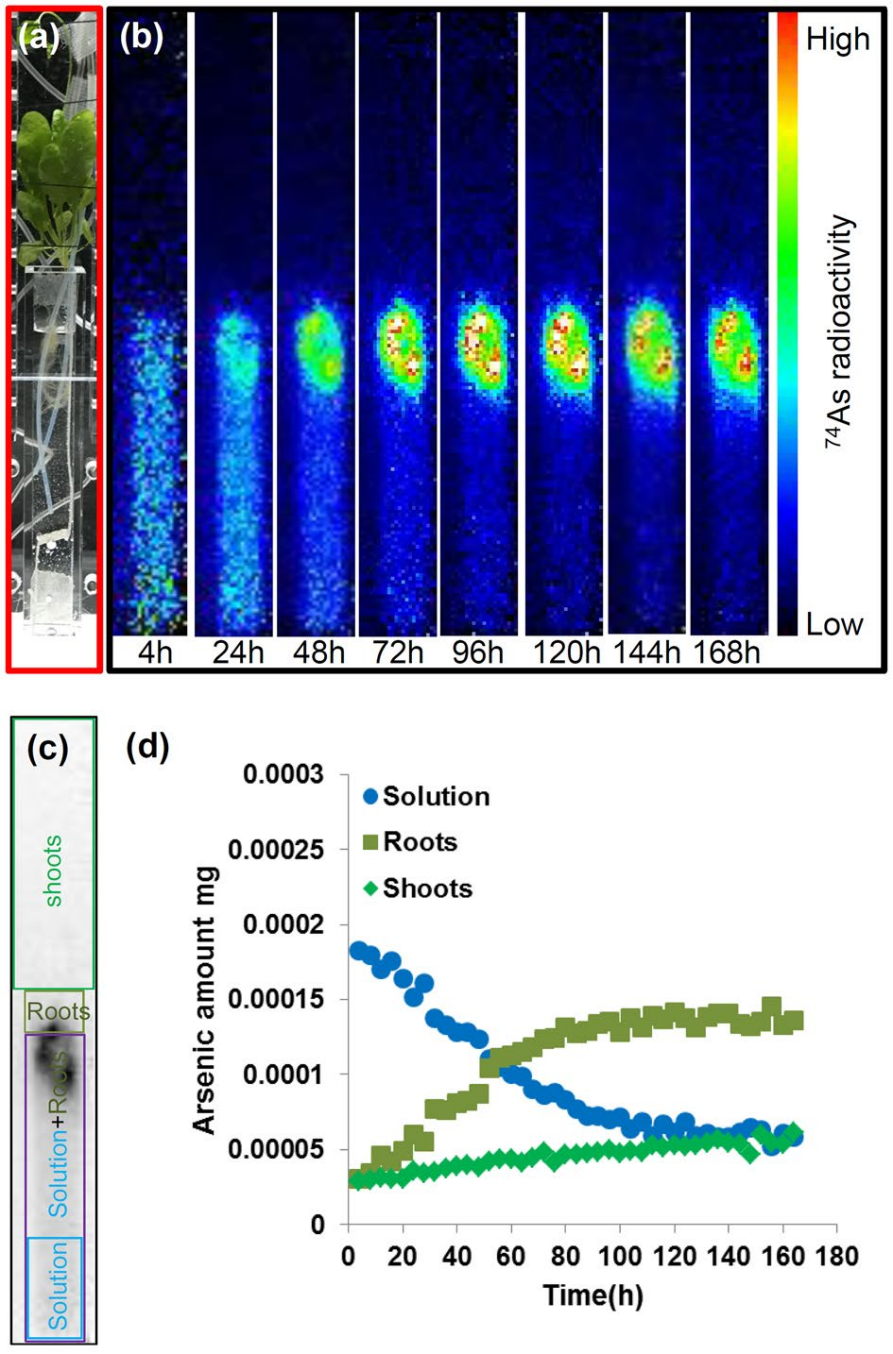
Serial images of ^74^As behavior and time course of the amount of As in different regions of *A*. *thaliana*. (**a**) Photograph of test plant in the experimental apparatus. The solid red rectangles indicate the field of view of the PETIS during the plant imaging. (**b**) Serial images of the *A*. *thaliana* (0–168 h). Each frame was created from the integration of original images collected every 5 min. (**c**) Examined regions in the plant. The blue rectangle indicates the region of the solution, the purple rectangle indicates the solution with the roots inside, the yellow-green rectangle indicates the roots above the solution and the green rectangle indicates the shoots. (**d**) The time course of the amount of As in the regions shown in (**c**) fed with 0.2 μM AsV including 2 kBq of ^74^As-labeled AsV.

To compare As accumulation kinetics of an As-hyperaccumulator and a non-As-accumulator, PETIS imaging was applied to a non-accumulator *A. thaliana* with 0.2 μM non-radioactive AsV including 2 kBq ^74^As-labeled AsV for 7 days (168 h) (Fig. 3a). Figure 3b and Supplementary video S2 show representative results of PETIS imaging of As translocation and accumulation in *A*. *thaliana*. Note that a rhizome does not exist in *A. thaliana*. The ROI of roots was observed as the bright spot in the PETIS imaging after 168 h (Fig. 3b,c). In *A. thaliana*, different from *P. vittata*, most of the As in the solution was retained in roots and there was little As translocation to shoots (Fig. 3d).

### Autoradiography analysis for *P*.* vittata* and *A*.* thaliana*

**Figure 4:**
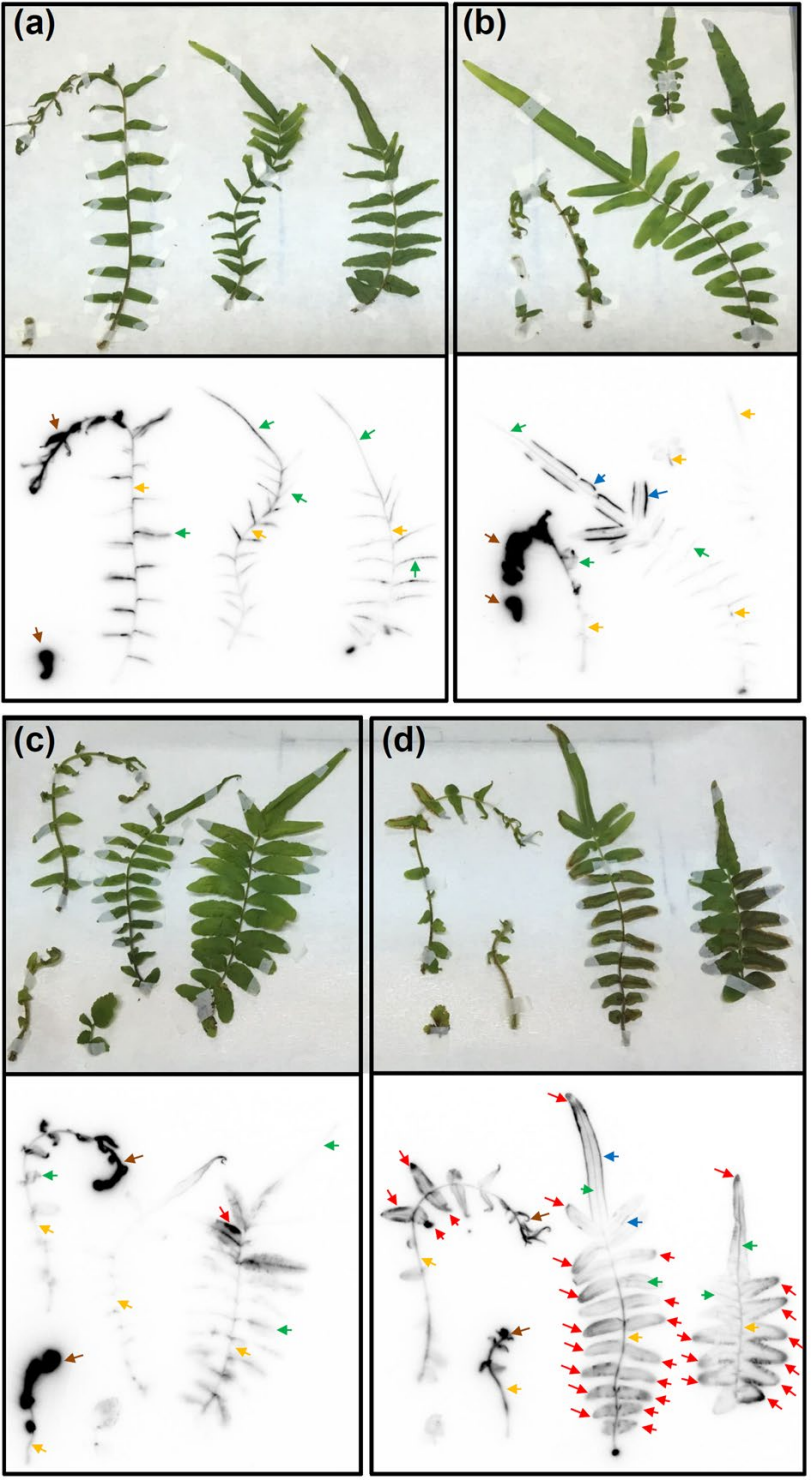
Autoradiography of static ^74^As in fronds of *P*. *vittata* supplied with As for 168 h. (**a**) Fed with 0.2 μM AsV. (**b**) Fed with 20 μM AsV. (**c**) Fed with 200 μM AsV. (**d**) Fed with 2000 μM AsV. All experiments included 2 kBq of ^74^As-labeled AsV. Fiddlehead and apical pinna of young fronds (brown arrow), midrib (green arrow), rachis (yellow arrow) sporangia (blue arrow) and the gray margin pinna showing necrotic symptoms (red arrow).

**Figure 5:**
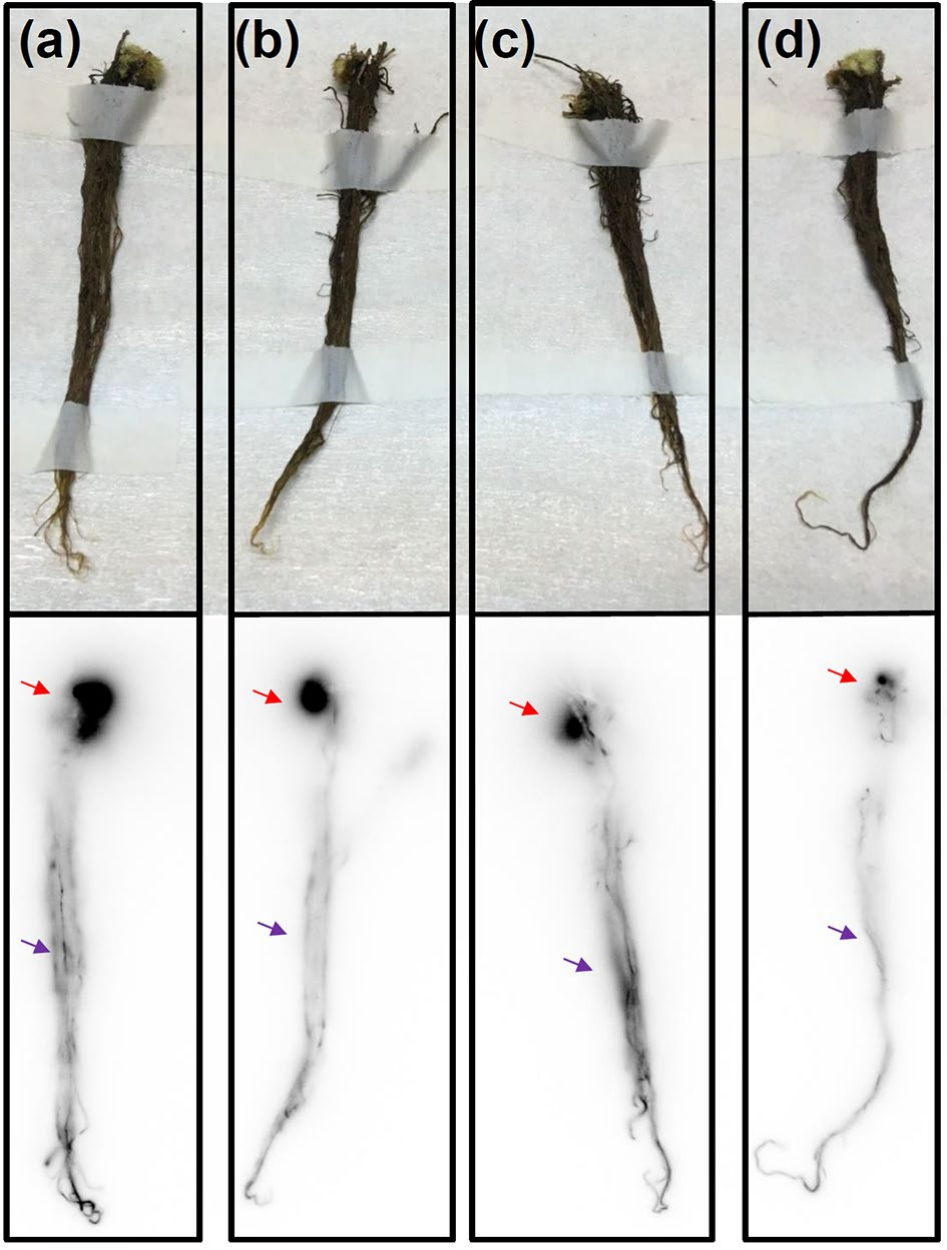
Autoradiography of static ^74^As in rhizomes and roots of *P*. *vittata* exposed to As for 168 h. (**a**) Fed with 0.2 μM AsV**.** (**b**) Fed with 20 μM AsV. (**c**) Fed with 200 μM AsV. (**d**) Fed with 2000 μM AsV. All experiments included 2 kBq of ^74^As-labeled AsV. Rhizomes (red arrow) and roots (purple arrow).

**Figure 6:**
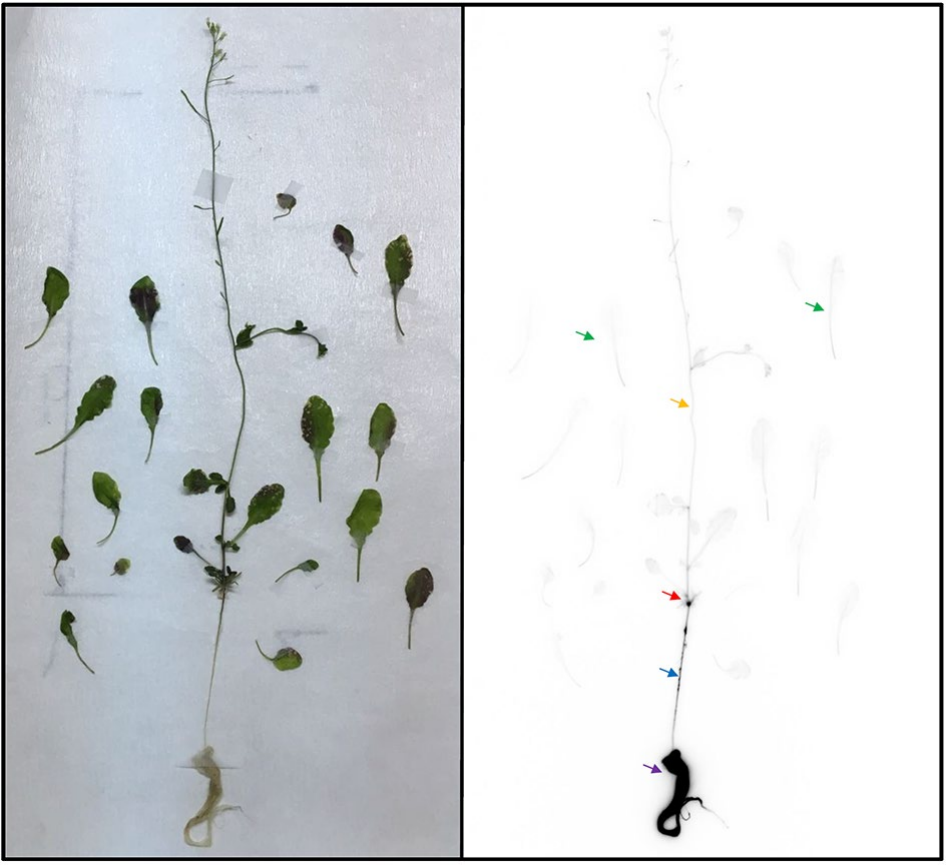
Autoradiography of static ^74^As in shoots and roots of *A*. *thaliana* exposed to 0.2 μM As including 2 kBq of ^74^As-labeled AsV for 168 h. Shoots (green arrow), main-stem (yellow arrow), shoots base (red arrow), primary root (blue arrow) and lateral roots (purple arrow).

After 168 h of PETIS experiments, plants were dissected and subjected to autoradiography of ^74^As. This provided the static distribution of As in individual fronds, rhizomes and roots for *P*. *vittata* (Figs. 4 and 5) and individual shoots, stem and roots for *A*. *thaliana* (Fig. 6), which was supplementary to the above PETIS analysis.

Figure 4a–d shows the fronds from *P. vittata* supplied with 0.2, 20, 200, and 2000 μM non-radioactive AsV as well as ^74^As, respectively (from left to right, young fronds to mature fronds in every panel). After 7 days of 0.2 and 20 μM As exposure, fronds did not show any necrotic symptoms but strong accumulation of ^74^As was observed in the fiddlehead and apical pinna of young fronds (brown arrow). Accumulation of As in the middle-to-basal pinna of young fronds and whole mature fronds were observed in the midrib (green arrow), rachis (yellow arrow) and sporangia (blue arrow) (Fig. 4a,b). After 7 days of 200 μM As exposure, the As accumulation pattern in fronds was similar to those of the 0.2 and 20 μM As treatments. However, necrotic symptoms (gray margin) were observed in one pinna of mature fronds (red arrow) and As accumulation was higher than others in mature fronds (Fig. 4c). After 7 days of 2000 μM As exposure, more pinna with necrotic symptoms were found in mature fronds and young fronds started to show necrotic symptoms (red arrow). The sections of pinna showing necrotic symptoms showed strong As accumulation (Fig. 4d). The accumulation of As in pinna with necrotic symptoms (gray margin) in this experiment was consistent with the phytofiltration experiment (Fig. 1d and Supplementary Fig. S1c), which showed that high As exposure induced the necrotic symptom because of the As accumulated in the margin of the pinna.

Figure 5a–d show the rhizomes and roots from *P*. *vittata* exposed to 0.2, 20, 200 and 2000 μM non-radioactive AsV as well as ^74^As, respectively. When compared with roots (purple arrow), strong accumulation of ^74^As in rhizomes (red arrow) was observed. The pattern of As accumulation in rhizomes from 0.2 μM to 2000 μM was confirmed by the PETIS imaging at 168 h (Fig. 2b).

Figure 6 shows individual shoots, stem and roots of *A*. *thaliana* exposed to 0.2 μM non-radioactive AsV as well as ^74^As. Strong accumulation of ^74^As was observed in the primary root (blue arrow), lateral root (purple arrow) and shoots base (red arrow). The As accumulation in these tissues was higher than in the main-stem (yellow arrow) and shoots (green arrow) in *A. thaliana*. These results showed that a non-accumulator *A. thaliana* took up As from the solution through the roots and translocated As to shoots through a shoot base, but most of the As was retained in the roots.

## Discussion

In this study, we used PETIS for the first time to obtain the noninvasive serial images of As behavior in living plants with positron-emitting ^74^As-labeled AsV. The As-hyperaccumulator *P*. *vittata* is efficient in AsV uptake, with the recent identification and investigation of a group of phosphate transporters in roots of *P*. *vittata* that were responsible for AsV uptake^4,5^. This is supported by the data of long term phytofiltration experiment and PETIS imaging presented here, which showed that AsV in solution was significantly decreased with different levels of AsV exposure (Figs. 1a and 2). After AsV enters the roots of *P*. *vittata*, most of it is translocated to rhizomes. AsV reductase in rhizomes played a critical role in reducing most of the AsV to AsIII within the rhizomes, and then most of the As (AsV and AsIII) was translocated to fronds via the xylem in *P*. *vittata*^8^. However, our data revealed that rhizomes in *P*. *vittata* not only translocated As to fronds but also accumulated the same high level of As as the fronds (Fig. 1c), and that As accumulation in rhizomes was not affected by the different concentrations of As applied (Figs. 2 and 5). Our data indicated that the function of rhizomes in *P. vittata* was As translocation and accumulation.

*Pteris vittata* fronds are well known for As accumulation. PETIS imaging and autoradiography analysis revealed that As accumulation in fronds shifted from young fronds to the gray margin of mature fronds, which contained high amounts of As with increasing As exposure (Figs. 2 and 4). This result was similar to that of a previous study of *P*. *vittata* cultivation in soil, which showed that under high As exposure, As concentrations in pinnae margins were higher than in the midrib, consistent with As-induced necrotic symptoms^15^. Unlike their work, our study showed that high As exposure induced necrotic symptoms in the pinna of *P*. *vittata* in two stages: gray margin and brown margin, where more As was accumulated in fronds brown margin than fronds gray margin and fronds new, and the As concentration in fronds brown margin was similar to that in rhizomes (Fig. 1c,d). These results indicated that after As was translocated from rhizomes to fronds, the As accumulation pattern in fronds was dependent on the level of As exposure, and increased As translocation to the margin of pinna in mature fronds might be regulated by rhizomes to protect the young fronds from high As exposure. In contrast, in the non-accumulator *A*. *thaliana*, most of the As was retained in the roots even with low As exposure (Figs. 3 and 6).

In this study, using a combination of a long term phytofiltration experiment and PETIS imaging and autoradiography analysis, we proposed the possible mechanism of As translocation and accumulation in *P*. *vittata*: After AsV is taken up by roots, most of the AsV is translocated to rhizomes and reduced to AsIII. Rhizomes in *P*. *vittata* keep accumulating As (AsV and AsIII) and regulating the As translocation to fronds. With low As exposure, most of the As is translocated and accumulated in young fronds. With high As exposure, As accumulation is shifted from young fronds to the mature fronds, especially in the margin of the pinna and induces the progression of necrotic symptoms from gray to brown.

## Methods

### Plant materials

*Pteris vittata* used in the present study was procured from Fujita Co., Ltd. (Tokyo, Japan). *Arabidopsis thaliana* ecotype Columbia (Col-0 accession) was kindly provided from Prof. Tomonobu Kusano (Graduate School of Life Science, Tohoku University).

Pre-cultivation of *P*. *vittata* seedling was conducted as described previously^25^. Three-month-old fern seedlings were individually transplanted into a hydroponic tank with surface area of 30 cm × 30 cm filled with 15 L of modified Hoagland nutrient solution (16 seedlings per tank). After four months of hydroponic cultivation, the 16-week As phytofiltration experiments including negative control (NC) were started (Supplementary Fig. S1a). Growth chamber conditions were set as a 14-h light period with a light intensity of 70 μmol m^−2^ s^−1^ and a 10-h dark period; 25 °C.

For the PETIS experiment, the size of the plant needs to be controlled, so three-month-old *P*. *vittata* seedlings were individually transplanted into a 250-mL vessel wrapped in aluminum foil containing 1/5 modified Hoagland nutrient solution. Plants were grown hydroponically for 2 months before the experiment. Seeds of *A. thaliana* were germinated, and then each plant was hydroponically grown for 2 months in a 250-mL vessel with 1/5 modified Hoagland solution in growth chamber with same condition for *P. vittata* before the experiment. Studies complies with local and national regulations.

### As phytofiltration experiment

The AsV concentration was adjusted to 30 mg/L by adding sodium arsenate (Na_2_HAsO_4,_ Wako Pure Chemical, Osaka, Japan) into 15 L Pi-free 1/5 modified Hoagland solution. Solution samples were collected at 0, 1, 2, 3, 6, and 16 weeks after the As exposure and the As concentration was measured. To investigate As accumulation in the fern, three ferns were collected (one plant from each tank) after 4 weeks of the exposure, and after 16 weeks of the phytofiltration experiment, the rest of the plants were harvested, and separated into fronds, rhizomes and roots.

As concentrations in the hydroponic solution and digested plant tissue samples were determined using an inductively coupled plasma mass spectrometer (ICP-MS) (ELAN 9000 or NexION 300, Perkin Elmer, Massachusetts, USA) as described previously^1^.

The values reported in both text and figures are the mean ± SE (standard error of the mean). The statistical significance (at 95% confidence) was tested using ANOVA.

### ^74^As tracer

^74^As radioisotope (T_1/2_ = 17.7 d) was produced as previously desccribed^26^. Ga_2_O_3_ powder(0.1 g) was prepared as a 10 mm diameter pellet. It was covered with aluminum foil (10 μm thickness) and used as a target. The target was irradiated with an alpha beam (30 MeV, 1.5 pμA) for 387 min from an AVF cyclotron at the Cyclotron and Radioisotope Center (CYRIC) in Tohoku University. The following processes for tracer preparation were started after the byproducts such as ^71^As (T_1/2_ = 65 h) and ^72^As (T_1/2_ = 26 h) decayed out at least 4 weeks after the irradiation. The irradiated Ga_2_O_3_ was dissolved in 1 mL of 12 M NaOH by heating up to 80 °C. After all the Ga_2_O_3_ was dissolved, about 2 mL of 6 M hydrochloric acid (HCl) was added to adjust the pH to ~ 6. The solution became a white suspension. The suspension was solvent extracted with 5 mL of 1 M Di-(2-ethylhexyl) phosphoric acid organic solution with hexane by shaking for 1 h. Ethanol was added to the aqueous phase after solvent extraction with hexane (about 3 mL) to make up the volume to about 50 mL, and sodium chloride (NaCl) in the solution was precipitated. For anion exchange column chromatography, 2 mL of Muromac 1 × 8 (100–200 mesh, Cl^–^ form) changed to OH^–^ form was used. The supernatant was charged into this column. Ethanol and contaminants were washed off by flushing 20 mL of H_2_O on the column. The As tracer was eluted with 8 mL of 0.1 M HCl. The eluate was neutralized with 0.8 mL of 1 M KOH (pH ~ 7). After that, the AsIII/AsV ratio after the dissolution of the target in H_2_O was determined by thin layer chromatography^27^. Ten μL of the solution was spotted about 1 cm from the end of a Si-60 thin layer on the Al plate. The plate was developed for 10 min by a mixture of 0.01 M aqueous sodium hydrogen L-tartrate /methanol solution in a ratio of 3/1 as mobile phase. The locations of the origin, AsIII, and AsV on the developed plate were then identified by Electronic Autoradiography (Instant-Imager, Packard Instruments). The retention factors (Rf) of 0.6 and 0.9 were used to specify AsIII and AsV, respectively. Only AsV was detected (Supplementary Fig. S2). ^74^As-labeled AsV was dissolved in an appropriate volume of the culture solution containing a designated concentration of nonradioactive As. Finally, 2 kBq ^74^As-labeled AsV (0.0074 pmol) with nonradioactive AsV was fed to each test plant.

### Whole-plant PETIS imaging

The PETIS imaging experiments were conducted following the methods of Fujimaki et al. and Suzui et al. with some modifications^16,21^. All imaging experiments were conducted in a clean booth (2 m × 2 m × 2 m) under controlled temperature and humidity conditions with continuous light at a density of 70 μmol m^−2^ s^−1^; 25 °C.

For *P*. *vittata*, the roots of intact *P*. *vittata* plants were inserted in an acrylic square container (KGS 1407-D01-00, Kumikouki Co., Gunma, Japan), and the fronds were sandwiched between 2 mm thick acrylic boards to maximize the annihilation of positrons. The acrylic board, which held four plants at a time, was placed in the field of view of the PETIS (PPIS-4800 positron imaging system; Hamamatsu Photonics, Hamamatsu, Japan) (Fig. 2a). Each container was supplied with 10 mL of Pi-free 1/5 Hoagland solution containing 2 kBq of ^74^As-labeled AsV, plus the different concentrations of non-radioactive AsV (as Na_2_HAsO_4_: 0.2, 20, 200, 2000 μM). The behavior of ^74^As in the plants, including the roots, rhizomes and fronds, was monitored by the PETIS every 5 min for 168 h. The solution was continuously stirred with gentle aeration to maintain a uniform composition in each compartment of the container.

Based on the PETIS imaging results of *P*. *vittata*, the PETIS imaging of A. thaliana was conducted with 0.2 μM AsV. The experimental conditions and preparation were the same as the PETIS imaging of *P*. *vittata*.

### Qualitative and quantitative analyses of PETIS data

In the PETIS imaging experiments, the indicated amounts of nonradioactive As were mixed with measured activities of pure ^74^As at a certain time before plant exposure. Therefore, the amount of total As (i.e. sum of radioactive and nonradioactive As) corresponding to the radioactivity of ^74^As at a given time can be easily determined. All the graphs and PETIS images shown in this paper indicated the determined amounts of total As, not just the intensities of the ^74^As signal. The time course data of the As amount (mg) in the regions of interest in the images were calculated by the values of the signal intensity (cps) extracted using the NIH Image J 1.50 software (http://rsb.info.nih.gov/ij/), counting efficiency of the system (cps Bq^−1^) and specific radioactivity (Bq mol^−1^).

### Autoradiography

The test plant was dissected and placed on paper sheets after the PETIS imaging was finished and then placed in contact with imaging plates (Fujifilm, Tokyo, Japan) in cassettes. After 2 days of exposure, the imaging plates were scanned using a bio-imaging analyzer (BAS-1500; Fujifilm, Tokyo, Japan) to obtain the auto-radiographic images of ^74^As in plant.

## Supplementary Information

Supplementary Figures.

Supplementary Video 1.

Supplementary Video 2.
