# Supplementary material for: New evidence of arsenic translocation and accumulation in Pteris vittata from real-time imaging using positron-emitting 74As tracer
Source: Sci Rep. 2021 Jul 8;11:12149. doi: 10.1038/s41598-021-91374-1 (PMC8263723; doi:10.1038/s41598-021-91374-1)
Supplement: Supplementary file 1 — Supplementary Figures. [file 41598_2021_91374_MOESM1_ESM.pdf]

Supplementary Information for

New evidence of Arsenic translocation and accumulation in *Pteris vittata* from real-time imaging using positron-emitting ^{74}As tracer

Yi Huang - Takeshi Kohda[1, 3✉], Zhaojie Qian[1], Mei-Fang Chien[1], Keisuke Miyauchi[2, 3], Ginro Endo[3], Nobuo Suzui[4], Yong-Gen Yin[4], Naoki Kawachi[4], Hayato Ikeda[5, 6], Hiroshi Watabe[5], Hidetoshi Kikunaga[6], Nobuyuki Kitajima[7] & Chihiro Inoue[1✉]

[1]Tohoku University, Graduate School of Environmental Studies, Miyagi 980-8579, Japan.

[2]Tohoku Gakuin University, Faculty of Engineering, Department of Civil and Environmental Engineering, Miyagi 980-8537, Japan.

[3]Tohoku Gakuin University, Research Institute for Engineering and Technology, Miyagi 980-8537, Japan.

[4]National Institutes for Quantum and Radiological Science and Technology (QST), Takasaki Advanced Radiation Research Institute, Gunma 370-1292, Japan.

[5]Tohoku University, Cyclotron and Radioisotope Center (CYRIC), Miyagi 980-8578, Japan.

[6]Tohoku University, Research Center for Electron Photon Science (ELPH), Miyagi 982-0826, Japan.

[7]Fujita Corporation, Technology Development Division, Kanagawa 243-0125, Japan.

🖂email: yi.huang.a3@tohoku.ac.jp, chihiro.inoue.b1@tohoku.ac.jp

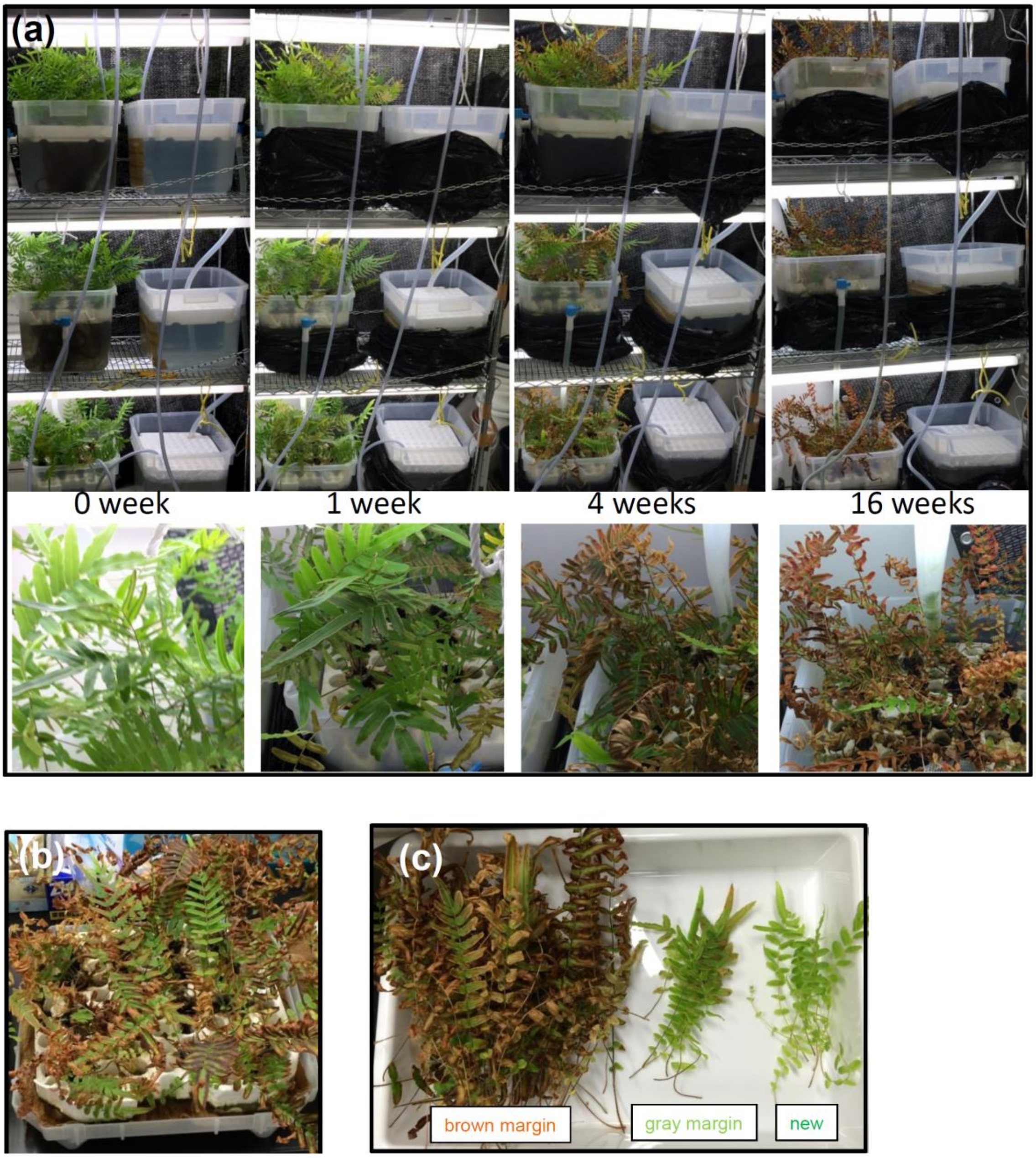

Fig. S1 Photographs of As accumulation in *P. vittata* during hydroponic cultivation with 30 mg/L AsV. Sixteen seedlings of *P. vittata* were planted in every tank (n=3), and then pre-cultured for 4 months before the hydroponics experiments of arsenic removal. (a) Upper line: Time course (0, 1, 4 and 16 weeks) of arsenic removal with *P. vittata* (left) and without *P. vittata* (right). Bottom line: Time course (0, 1, 4 and 16 weeks) of

changing pinna margin color in fronds of *P. vittata*. After 16 weeks, all plants (three tanks of *P. vittata*) were harvested and separated into frond, rhizome and roots for arsenic analysis. (b) One tank of *P. vittata*. (c) Separation of fronds from (b) into fronds brown margin, fronds gray margin and fronds new.

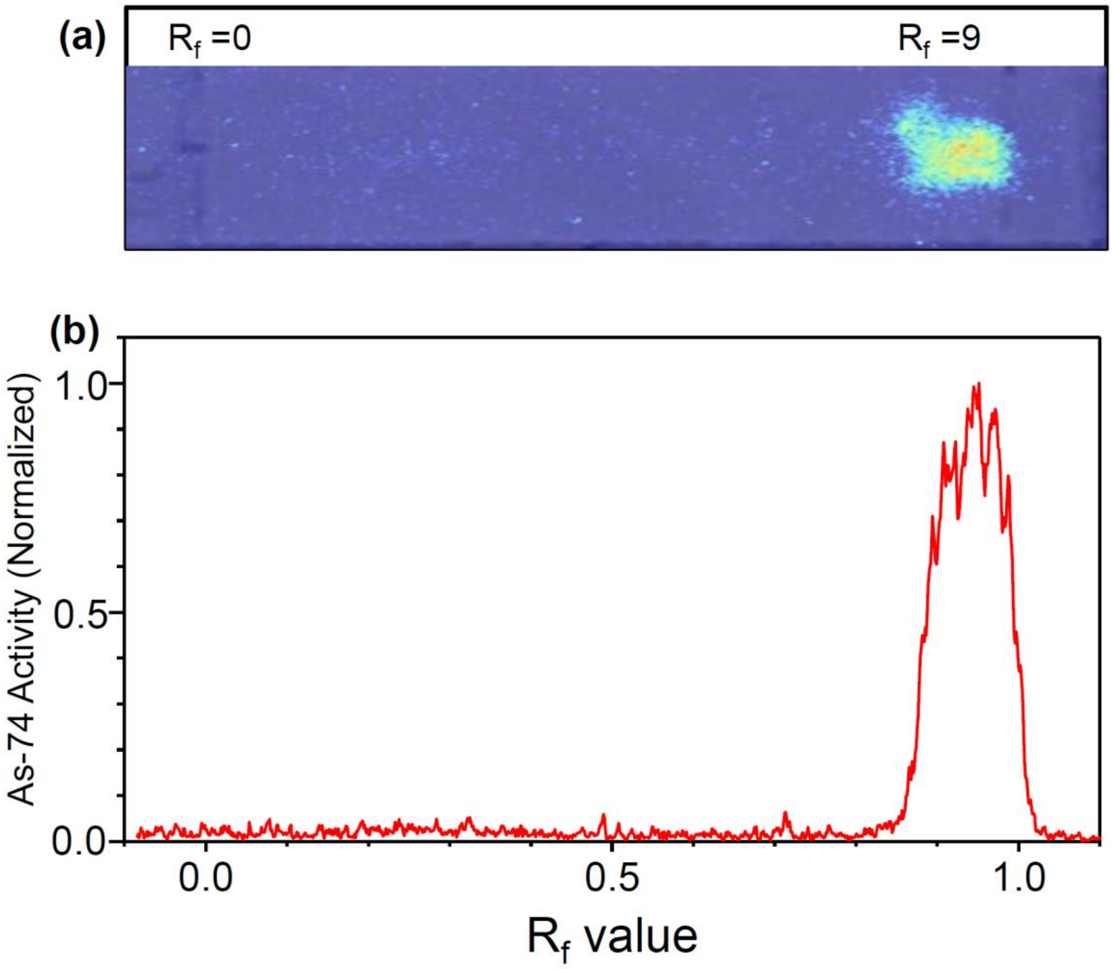

Fig. S2 Activity profile of ^{74}As used in this study. **(a)** Image of thin layer chromatography (TLC) plate showing distribution of the activity of ^{74}As for this study. The starting point R_f=0 and arsenate (AsV) retardation point R_f=0.9 were shown. **(b)** Electronic autoradiography of the TLC plate. The counts of ^{74}As radioactivity from R_f=0.9 was normalized to 1.0.
